# Real-time prediction of intradialytic relative blood volume: a proof-of-concept for integrated cloud computing infrastructure

**DOI:** 10.1186/s12882-021-02481-0

**Published:** 2021-08-09

**Authors:** Sheetal Chaudhuri, Hao Han, Caitlin Monaghan, John Larkin, Peter Waguespack, Brian Shulman, Zuwen Kuang, Srikanth Bellamkonda, Jane Brzozowski, Jeffrey Hymes, Mike Black, Peter Kotanko, Jeroen P. Kooman, Franklin W. Maddux, Len Usvyat

**Affiliations:** 1 Fresenius Medical Care, Global Medical Office, 920 Winter Street, Waltham, MA 02451 USA; 2 Maastricht University Medical Center, Maastricht, The Netherlands; 3 Fresenius Medical Care North America, Waltham, MA USA; 4 Renal Research Institute, New York, NY USA; 5 Icahn School of Medicine at Mount Sinai, New York, NY USA

**Keywords:** End Stage Kidney Disease, Real-Time Prediction, Machine Learning

## Abstract

**Background:**

Inadequate refilling from extravascular compartments during hemodialysis can lead to intradialytic symptoms, such as hypotension, nausea, vomiting, and cramping/myalgia. Relative blood volume (RBV) plays an important role in adapting the ultrafiltration rate which in turn has a positive effect on intradialytic symptoms. It has been clinically challenging to identify changes RBV in real time to proactively intervene and reduce potential negative consequences of volume depletion. Leveraging advanced technologies to process large volumes of dialysis and machine data in real time and developing prediction models using machine learning (ML) is critical in identifying these signals.

**Method:**

We conducted a proof-of-concept analysis to retrospectively assess near real-time dialysis treatment data from in-center patients in six clinics using Optical Sensing Device (OSD), during December 2018 to August 2019. The goal of this analysis was to use real-time OSD data to predict if a patient’s relative blood volume (RBV) decreases at a rate of at least − 6.5 % per hour within the next 15 min during a dialysis treatment, based on 10-second windows of data in the previous 15 min. A dashboard application was constructed to demonstrate how reporting structures may be developed to alert clinicians in real time of at-risk cases. Data was derived from three sources: (1) OSDs, (2) hemodialysis machines, and (3) patient electronic health records.

**Results:**

Treatment data from 616 in-center dialysis patients in the six clinics was curated into a big data store and fed into a Machine Learning (ML) model developed and deployed within the cloud. The threshold for classifying observations as positive or negative was set at 0.08. Precision for the model at this threshold was 0.33 and recall was 0.94. The area under the receiver operating curve (AUROC) for the ML model was 0.89 using test data.

**Conclusions:**

The findings from our proof-of concept analysis demonstrate the design of a cloud-based framework that can be used for making real-time predictions of events during dialysis treatments. Making real-time predictions has the potential to assist clinicians at the point of care during hemodialysis.

## Background

Hemodialysis (HD) involves removal of fluid from the circulating blood by ultrafiltration and refilling from the extravascular compartments [1]. This process helps preserve blood pressure and tissue perfusion [2]. However, inadequate refilling could lead to a variety of intradialytic symptoms, such as intradialytic hypotension (IDH), fatigue, and cramping [3, 4]. IDH can lead to cardiac complications and an increased risk of death [5–8].

Studies have shown the role of relative blood volume (RBV) and how adapting the ultrafiltration rate has a positive effect on intradialytic symptoms [9, 10]. However, it has been clinically challenging to identify changes in RBV in real time to proactively intervene and reduce potential negative consequences of volume depletion. Hence leveraging advanced technologies to process large volumes of dialysis and machine data in real time and developing prediction models using machine learning (ML) is critical in identifying these signals.

A network of dialysis clinics routinely captured hematocrit, oxygen saturation, and intravascular blood volume during dialysis using Optical Sensing Device (OSD) device [11–13]. The OSD provides clinicians with the ability to have near real-time monitoring of the patient’s clinical status during HD. During dialysis treatments, data is collected every ten seconds, which is required to be stored, curated, and analyzed timely interventions. There is a dearth of knowledge about utilizing this machine data for monitoring treatment level parameters and personalizing care for HD patients. This may be secondary to traditional storage and computing resources being unable to handle the processing of such large data stores.

Big data technologies and cloud-based services are novel tools that can provide the necessary infrastructure to support such near real-time applications. Big data is a field that incorporates ways to analyze, systematically extract information from, or otherwise deal with data sets that are too large or complex to be dealt with by traditional software [14]. Cloud technology moves big data processing off local computers and onto shared web services, allowing for greater optimization of resources and faster processing as a result. Cloud platforms provides a secure, efficient, and reliable way to process and analyze data.

We conducted a retrospective analysis to assess dialysis treatment data from 2019. This analysis was used to develop a proof-of-concept that cloud infrastructure can be used in clinical care and provide necessary data to consider if implementation in the future is warranted. The model developed in this proof-of-concept was not utilized in clinical practice.

We created a ML application to identify patients at risk of having their RBV decrease at a rate of at least − 6.5 % per hour anytime during HD. A dashboard application was constructed demonstrate how reporting structures may be developed to alert clinicians in real time of at-risk cases.

## Methods/design

### General design

 For this proof-of-concept analysis, we used data from adult patients treated at six clinics (Fresenius Kidney Care, Waltham, MA, United States) that universally used OSD during HD as a standard of care between December 2018 through August 2019. In these six clinics, there was hardware previously setup to transfer data from the OSD device to a secure Internet of Things (IoT) private server on Amazon Web Services (AWS; Amazon Web Services, Inc., Seattle, WA, United States) using IoT software [15, 16]. The AWS server was compliant with the Health Insurance Portability and Accountability Act (HIPAA) [15]. Amazon Web Services (AWS), Microsoft Azure, and Google’s cloud platforms are the most broadly adopted web services platform in the world [17–19].

The goal of this analysis was to use historic OSD data to build a prediction model that can actively classify patients at risk of having their RBV decrease at a rate of at least − 6.5 % per hour within the next 15 min of HD throughout the entire treatment. Also, we aimed to construct a dashboard to that could be considered for delivery of alerts for patients predicted at risk.

This analysis was performed under a protocol that was approved by New England Institutional Review Board under a waiver of informed consent per title 45 of the United States Code of Federal Regulations part 46.116(f) (Needham Heights, MA, United States; NEIRB# 17-1311567-1). The analysis was conducted in adherence with the Declaration of Helsinki.

### Patient population

We included data from patients who were greater than or equal to 18 years of age and females were not known to be pregnant.

### Optical sensing device

The OSD (Crit-Line®, Bad Homburg, Germany) profiles patient’s intradialytic status to assist clinicians monitor the treatment assessment and intervention during hemodialysis [20]. By monitoring blood volume percent changes, caregivers can adjust treatment as necessary to maximize fluid removal and prevent common intradialytic symptoms, such as IDH, nausea, vomiting, and cramping [9, 21–24], as well as minimize the risk of worse outcomes [12, 25].

Per the manufacture’s specifications for RBV thresholds [20], when the rate of change in RBV, based on the latest 15 min of data, is decreasing less than − 3 % per hour, the ultrafiltration rate might be increased without immediate risk of intradialytic symptoms. In this case the patient’s plasma refill rate is occurring at the same or a greater rate than the ultrafiltration rate. When the rate of change in RBV, based on the latest 15 min of data, is decreasing between − 3 and − 6.5 % per hour, it indicates a suitable compromise between a high ultrafiltration rate and the prevention of intradialytic symptoms. When the rate of change in RBV, based on the latest 15 min of data, is greater than − 6.5 % per hour, there is a rapid decrease in RBV and bears a higher risk for intradialytic symptoms, such as lightheadedness, nausea, vomiting, cramping, or hypotension. Prior studies have shown reductions in intradialytic complications with ultrafiltration based on RBV targets in relatively consistent ranges [9, 21–24], and that ultrafiltration performed targeting RBV deceases between − 3 and − 6.5 % per hour associates with better patient outcomes [12, 25].

### Model data and features

The ML model was trained and tested on a static set of historical observations from our system. Data was derived from three sources: (1) OSDs, (2) HD machines, and (3) patient electronic health records.

OSD data and treatment data from the 2008 T® dialysis machines were collected every 10 s during dialysis treatments. OSD data included variables like blood volume alert level, RBV, changes in hematocrit, hemoglobin, oxygen saturation, minimum oxygen saturation, and oxygen alert level.

Dialysis machine data included variables such as systolic blood pressure, diastolic blood pressure, mean arterial blood pressure, pulse, delivered equilibrated (e)Kt V, average small molecular clearance [Kecn], projected single pool (sp) Kt V, first plasma Na, body volume, blood flow rate, conductivity, dialysate flow rate, intervention performed on the machine, arterial pressure, dialysate temperature, venous pressure, ultrafiltration rate, blood volume processed, ultrafiltration goal, ultrafiltration volume removed, and remaining time on dialysis in minutes (RTD).

Patient demographic information such as age, height, access type, and clinic ID were referenced from the on-premises clinical data warehouse. Patient measures in the clinic on the day of treatment included pre-dialysis/post-dialysis weight and the type of dialyzer used in treatment.

OSD and 2008T® dialysis machine data from five separate time windows: 1, 5, 10, 15 min, and since-start-of-treatment windows were used to derive additional intra-treatment features using average, minimum, maximum and standard deviation for each time window. The final dataset spanned 751,354 treatment records and 493 input variables including features for average, minimum, maximum, and standard deviation for the continuous variables at each time point.

### Predictive model

**Fig. 1:**
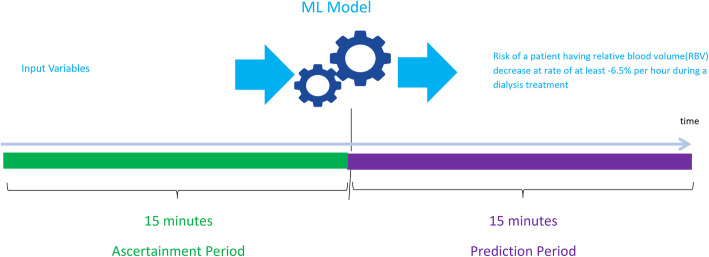
Ascertainment Period and Prediction Period

The model was built using the AWS Sagemaker [26] development platform. The curated final dataset of 751,354 treatment records was randomly split into training data (80 %), validation data (10 %) and test data (10 %). The target variable was a binary indicator of patients who experienced a decrease in RBV at a rate of at least − 6.5 % per hour during a dialysis treatment within the next 15 min. Figure 1 shows the ascertainment period and the prediction period for the model.

The data showed a 22 % prevalence within observations in which RBV decreased at rate of at least 6.5 % per hour during a dialysis treatment. Given the imbalanced nature of our data, we limited our ML model selection to algorithms that deal well with such data, including support vector and random forest families [27, 28]. The final model was trained using a ML tool known as an extreme gradient boosting (XGboost) algorithm [29]. Hyperparameters are model-specific internal parameters that are initially set to certain default values to cover general use cases. These parameters must be tuned for the problem at hand to get optimal model performance [30]. After model selection, the Bayesian optimization strategy implemented by Amazon SageMaker was used to tune the model hyperparameters to maximize the AUROC [31]. In the Bayesian tuning strategy ML algorithms performance is modeled as a sample of a Gaussian process [32, 33]. This allows information from prior iterations to inform the next parameters to try to optimize model performance, balancing both exploration of values not yet used with exploitation of the best-known results.

The predicted probability output by the model was converted to a binary prediction to predict positive and negative cases of RBV decreasing at rate of at least − 6.5 % per hour during a dialysis treatment. The cutoff threshold for the binary prediction was set to 0.08, so if the prediction score was above 0.08, then the patient was flagged to be at risk of decreasing RBV. The threshold was set by evaluating the results of the training and validation data.

Feature importance from the gradient boosting algorithm was used to derive top features (variables) that were considered highly predictive of the outcome. The feature importance is calculated using the gain method, or the relative contribution of the corresponding feature for each tree in the model. The method works by averaging the training loss reduction caused by feature utilization for each split in the decision tree [34].

### Conceptional analysis design

**Fig. 2:**
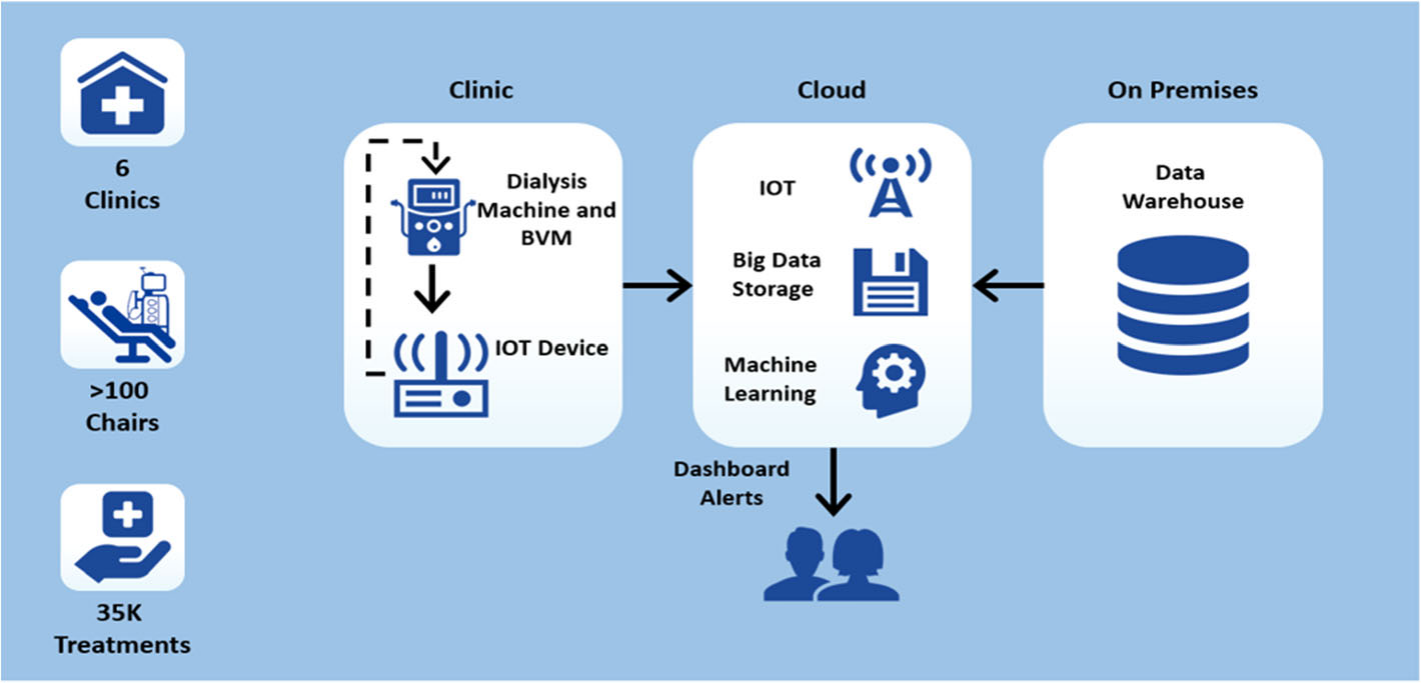
Analysis Design

Figure 2 shows a general design of the analysis setup. Conceptually, the analysis consisted of three main components: (1) Hardware and devices needed to monitor patients, (2) Cloud-based Service for real-time data analysis and communication, and (3) On-premises secure Data Warehouse to reference patient-protected information needed for data analysis.

In the cloud, IoT software processed incoming data from the clinic. The data was then curated into a big data store within the cloud. The big data store referenced on-premises data warehouse to securely extract patient-protected information and other clinical data and then to securely feed into the ML model. These multiple sources of data were made available to the Machine Learning Engine (MLE) which was also hosted in the cloud. The MLE would then make a prediction based on the data and generate an alert to the clinicians and nurses for identified at-risk cases. The entire analysis pipeline had to be optimized to ensure low latency (i.e. ensure timeliness of near real-time prediction). This optimization process is beyond the scope of the current discussion.

### Cloud computing infrastructure flow for generating real time dashboard

**Fig. 3:**
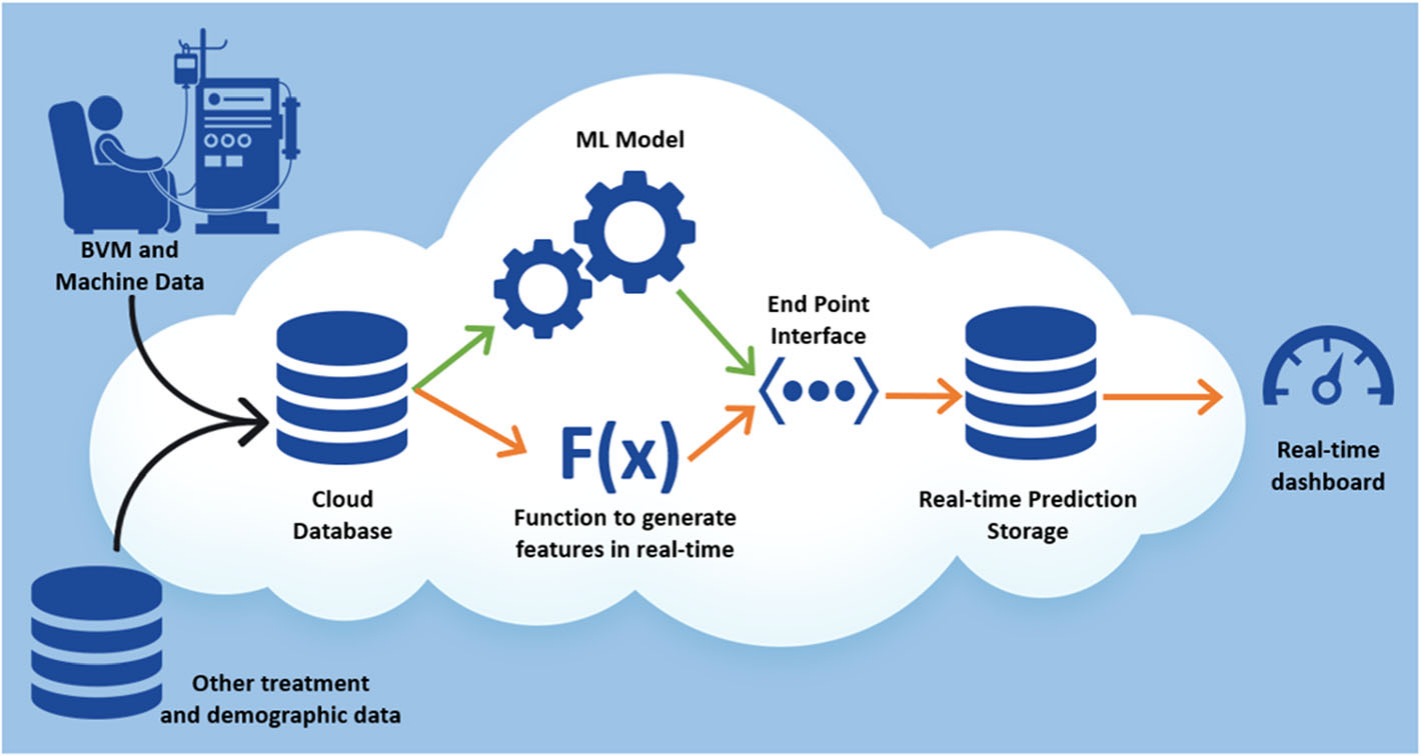
Data Flow for Real-time prediction using the AWS Cloud Environment

The data flow for the entire modeling pipeline within AWS is shown in Fig. 3. Green arrows in Fig. 3 show how the data flows from the clinics using OSD, the dialysis machines, and the warehouse into the cloud to train a model and provide data to the endpoint interface. The orange arrows show how the data flows in real time from the clinic using OSD and the data warehouse. Each new message in the cloud data store triggers a function, which creates 493 different features used in the trained model. These features are then provided as an input data parameter to the endpoint interface to generate a prediction and store the results in another data store. The prediction results are then used in a dashboard to generate a proof-of-concept clinical user interface.

### Analysis of ML model performance

Performance of ML model was measured by the area under the receiver operating curve (AUROC) in the training, validation, and testing datasets, as well as the recall and precision in the testing datasets. AUROC measures the rate of true and false positives classified by the prediction model across probability thresholds. Table 1 shows the definition of true/false positive and negative predictions classified by the model in the assessment of performance in the testing dataset.

**Table 1:** Definition of true/false positive and negative predictions classified by the model in the assessment of performance in the testing dataset

True positives	Patients correctly classified as having a risk of their relative blood volume (RBV) decrease at a rate of at least − 6.5 % per hour within the next 15 min by the model.
False positives	Patients incorrectly classified as having a risk of their RBV decrease at a rate of at least − 6.5 % per hour within the next 15 min by the model.
True negatives	Patients correctly classified as not having a risk of their RBV decrease at a rate of at least − 6.5 % per hour within the next 15 min by the model.
False negatives	Patients incorrectly classified as not having a risk of their RBV decrease at a rate of at least − 6.5 % per hour within the next 15 min by the model.

Recall (sensitivity) measures the rate of true positives classified by the model at a specified threshold and is calculated as follows:

$$\normalsize \normalsize Recall = \normalsize \frac{number\ of\ true\ positives}{ (number\ of\  true\ positives\ +\ number\ of\ false\ negatives )}$$

Precision measures the positive predictive value for the model at a specified threshold and is calculated as follows:

$$\normalsize Precision = \normalsize \frac{number\ of\ true\ positives}{ (number\ of\ true\ positives\ +\ number\ of\ false\ positives )}$$

Similarly, Specificity for the model is defined as:

$$\normalsize Specificity = \normalsize \frac{number\ of\ true\ negatives}{ (number\ of\ true\ negatives\ +\ number\ of\ false\ positives)}$$

And the Negative Predictive Value (NPV) is defined as:

$$\normalsize NPV = \normalsize \frac{number\ of\ true\ negatives}{ (number\ of\ true\ negatives\ +\ number\ of\ false\ negatives )}$$

AUROC, recall, precision, specificity and NPV metrics yield scores on a scale of 0 (lowest) to 1 (highest). A model performing at chance would yield an AUROC of 0.5. The cutoff threshold for classifying predictions was selected to optimize recall and precision according to the use case.

## Results

### Patient characteristics

 We obtained data from 616 adult in-center HD patients that were treated in six clinics to build the prediction model. Patient demographics are shown in Table 2. The descriptive statistics of numeric input variables used to train the model are shown in Table 3.

**Table 2:** Demographics of patients at the start of the study period (entire cohort)

Patient Characteristics	Value
Number of Patients	616
Average Age	64.5(SD: ±14.83)
Male	57.8 %
Black	27.5 %
White	68.6 %
Hispanic	22.1 %
Congestive Heart Failure	24.7 %
Diabetes	39.1 %
Hypertension	77.5 %
Ischemic Heart Disease	24.5 %
Average Albumin [g/dL]	3.8(SD: ±0.38)

**Table 3:** Descriptive Statistics of Numeric Input Variables (Training Data)

Variable	N	Mean ± SD
Diastolic Blood Pressure [mmHg]	562,012	70.13 ± 14.42
Mean Arterial Blood Pressure [mmHg]	562,012	96.72 ± 18.13
Mean Pulse [bpm]	562,012	71.83 ± 11.08
Systolic Blood Pressure [mmHg]	562,012	133.75 ± 24.27
Delivered equilibrated (E)Kt V	415,498	0.62 ± 0.24
Mean Kecn	539,626	252.38 ± 33.25
Projected single pool (sp)Kt V	533,226	0.78 ± 0.54
First Plasma Na [mEq/L]	537,453	140.24 ± 3.78
Body Volume [L]	533,226	34.84 ± 8.46
Critline Relative Blood Volume Alert [%]	601,210	-12.42 ± 3.95
Relative blood volume (RBV) [%]	601,210	-4.16 ± 6.87
Changes in hematocrit [%]	601,210	26.83 ± 15.42
Hemoglobin [g/dL]	601,210	8.96 ± 5.33
Oxygen saturation [%]	601,210	69.39 ± 39.25
Minimum Oxygen Saturation [%]	601,210	88.37 ± 16.84
Oxygen Alert Level [%]	601,210	68.29 ± 34.64
Blood Flow Rate [mL/min.]	601,210	348.99 ± 142.23
Conductivity [mS/cm]	601,210	13.7 ± 1.09
Dialysate Flow Rate [mL/min.]	601,210	643.03 ± 182.94
Monitor Temp [°C]	601,210	36.49 ± 0.88
Arterial Pressure [mmHg]	601,210	-162.43 ± 77.12
Dialysate Temperature [°C]	601,210	32.86 ± 52.96
Venous Pressure [mmHg]	601,210	156.84 ± 71.85
Ultrafiltration Rate [mL/Hr]	601,210	550.6 ± 350.28
Blood Volume Processed [L]	601,210	420.41 ± 271.47
Remaining Time on Dialysis [Min]	601,210	95.46 ± 71.16
Ultrafiltration Goal [mL]	601,210	2506.46 ± 1042.49
Ultrafiltration Volume Removed [mL]	601,210	1280.32 ± 999.72
Age [Years]	560,899	66.57 ± 14.35
Height [cm]	535,433	168.1 ± 11
Most Recent Post-Dialysis Weight [Kg]	552,865	80.12 ± 24.05
Most Recent Pre-Dialysis Weight [Kg]	552,865	82.08 ± 24.55
Average 30 days post dialysis weight [Kg]	552,865	80.06 ± 24.08
Average 30 days pre dialysis weight [Kg]	552,865	82.03+/-24.59

*SD *Standard Deviation

### ML Model performance and feature importance

The resulting predictive model was tested on 10 % (75,072 records) of the treatment data from all 616 patients, which was withheld during training.

**Fig. 4:**
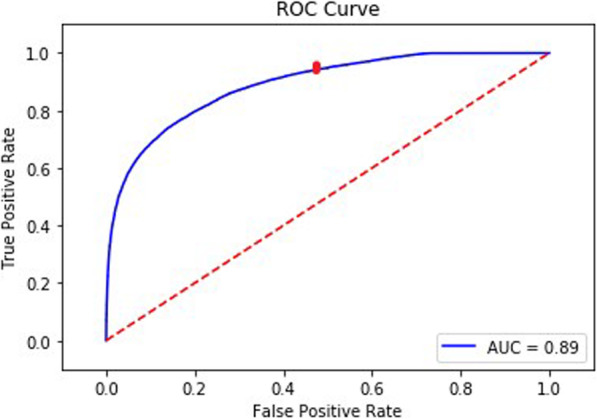
Area Under the Receiver Operating Curve for the Prediction Model

Using a low threshold of 0.08, the model had a recall rate of 0.94, meaning the model was able to capture 94 % of the observations that had a decrease in RBV at a rate of at least − 6.5 % per hour within the next 15 min. The precision of the model was 0.33. The specificity for the model was 0.52 and the NPV was 0.97. The AUROC (Fig. 4) for the final hyperparameter tuned model was 0.89. The red dot on the figure shows the true positive rate and the false positive rate at a threshold of 0.08.

**Fig. 5:**
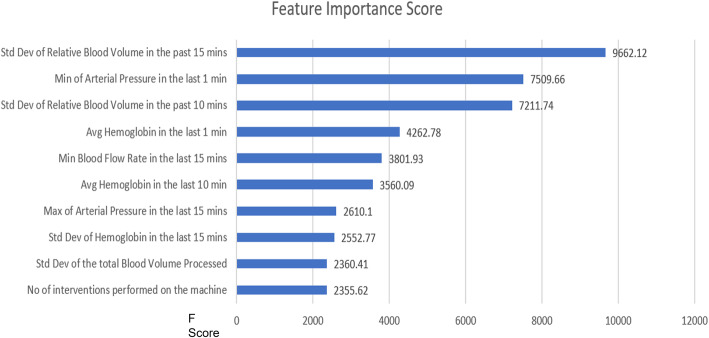
Top 10 features from Prediction Model and the Feature importance Score

Figure 5 shows a list of the top 10 features from the tuned model that were most predictive of a patient experiencing their RBV decrease at a rate of at least − 6.5 % per hour during a dialysis treatment in the next 15 min. It shows how valuable each feature was to the model in predicting the outcome. Higher value of the feature implies it is more important in calculating the outcome of the model.

### Proof-of-concept dashboard

**Fig. 6:**
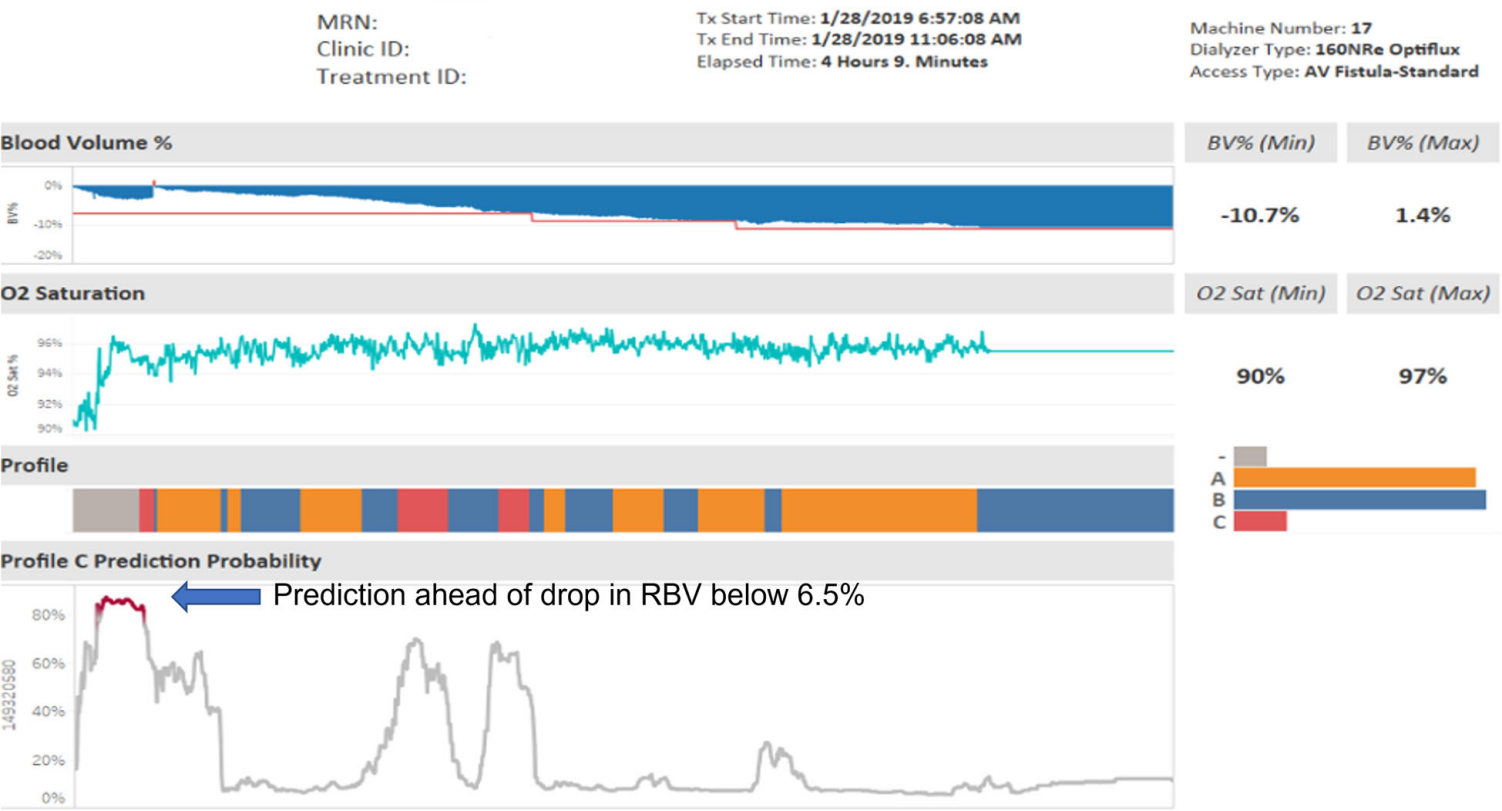
Proof-of-Concept Dashboard for Monitoring Risk during Dialysis Treatment

Figure 6 shows the proof-of-concept dashboard for a patient during dialysis treatment. The patient goes through various stages of having a risk of RBV decreasing at rate of at least − 6.5 % per hour (that is entering Profile C as shown in the figure). The probability of the prediction of profile C generated from the model is above 80 % before the actual occurrence of the event denoted in red under the Profile header. The RBV % at this point drops below − 6.5 % as shown under the Blood Volume % header. This dashboard illustrates that the model was able to predict the occurrence of the event before it happened.

## Discussion

The findings from our proof-of-concept analysis suggest the potential for real-time reporting and prediction of treatment blood volume profiles that are associated with an increased risk of intra-dialytic symptoms and would subsequently be amenable to intervention. Furthermore, the architectural framework demonstrated in this paper can be used for making real-time predictions of other events during dialysis treatments; and as such this analysis serves as a proof-of-concept. Making real-time predictions can help clinicians and nurses to provide proactive support at the point of care during dialysis treatment. A practical implication for the present would be that, if nurses and clinicians are alerted to the risk of a drop in the blood volume 15 min prior to the RBV decreasing at rate of at least − 6.5 % per hour during a dialysis treatment, they would have ample time to intervene and adjust the ultrafiltration rate in order to prevent that patient from entering the risk zone for intradialytic symptoms like IDH [20].

Prior studies have been attempted to monitor hematocrit and reduce intradialytic symptoms, however, they were not used in standard practice because of the difficulty in interpreting the OSD outputs updated every 10 s [9, 35]. The ML model presented in this analysis enhances the findings and delivers them in a comprehendible way. The top predictors of a RBV decreasing at a rate of at least − 6.5 % per hour were shown to include the variability in RBV in the prior 10 and 15 min, minimum arterial pressure in the prior 1 min, mean hemoglobin in the prior 1 and 10 min(s), and minimum blood flow rate in the prior 15 min, as well as other metrics related to atrial pressure, hemoglobin, and total blood volume processed (Fig. 5). The feature importance of these parameters appears to be identifying combinations of minor signals providing early signs of issues with ultrafiltration (e.g. peristaltic pump being starved of flow due to higher resistance in the access circuit). Ultimately, this model may have the potential to support the clinicians by classifying risk levels in near real-time. This analysis also adds onto the proof-of-concept analysis from Barbieri et al., where they developed an artificial neural network model predicting session-specific Kt/V, fluid volume removal, heart rate, and BP based on patient characteristics, historic hemodynamic responses, and dialysis-related prescriptions [36, 37].

Cloud Computing Resources provide seamless tools to build, analyze, and integrate real-time predictive models without investing in many hardware and software resources on premise. This allows for a secure and cost-effective way of building predictive models when resources are limited. These applications can also be scaled on-demand, where support can be expanded from tens to hundreds of clinics seamlessly.

Along with the disease burden, inadequate dialysis process may play a role in the pathophysiology of cardiac injury, cognitive impairment, and brain injury in HD patients [38–40]. Large amounts of data collected from the dialysis machines to build and deploy ML models can be used in personalizing dialysis treatments for HD patients. Optimizing dialysate temperature, monitoring access flows, modeling retention solute clearance and electrolyte profiling, and predicting IDH are other examples of how machine data can be utilized to personalize treatments for patients. Successful applications of analyzing and modeling large amounts of clinical data from the machines will require technology and a framework like what has been presented in this paper.

This paper provides an important proof-of-concept for the application of a ML-based model in the prevention of intradialytic complications. However, it should be stressed that while the decline in RBV during dialysis is an important risk factor for IDH, the critical decline in RBV and the level at which the patient experiences IDH also differs significantly between patients [7, 21]. IDH is an important risk factor for mortality, as well as for ischemia of vital organs, such as heart and brain, which may lead to long-term organ damage. Therefore, methods to reduce the risk for this complication are of vital importance [25, 40–42]. Other factors, such as an impairment in vascular reactivity or the cardiovascular status of the patient play an important role in the sensitivity of the patient to a decline in RBV. Moreover, there is a possibility of misclassification of patients at risk, where the model predicts that the RBV will decrease at a rate of at least − 6.5 % per hour during a dialysis treatment whereas it does not; hence the clinical intervention should be designed in such a way that it does not have an adverse impact on the treatment or the patient. In this respect, it is also important that profiles with a small decline in RBV may carry the risk of adverse outcomes, possibly because of its relationship with fluid overload [25]. Therefore, the results of the model should always be interpreted in the context of the patient.

The goal of this proof- of-concept project was to demonstrate the architecture of how machine data can be utilized in real time. The goal of the dashboard if implemented is to capture as many patients as possible who would have an adverse intradialytic event or have the risk of dropping RBV at the rate of at least − 6.5 % per hour. Hence, the focus was on sensitivity rather than specificity or precision when determining the threshold used to evaluate model performance. However, in a real-world implementation, the optimal threshold can be selected to minimize either false positives or false negatives, which will depend on the intervention and reporting demands.

This architecture also demonstrates the capabilities of a cloud-based framework in handling the large amounts of patient and treatment data collected from dialysis machines. ML models can be utilized for personalizing care in dialysis patients in real time. However, there will be instances when the ML model will predict incorrectly, so teams developing interventions using ML models need to be aware of this limitation. This proof-of-concept could also be used for predicting low or differing ranges of RBV. The clinical team responsible for designing interventions will need to interpret RBV targets and adjust ultrafiltration rate in a personalized manner considering each patient’s unique history of intradialytic complications. Also, the true performance of the ML model can only be demonstrated after conducting randomized clinical trials. The cloud-based framework should allow scaling of this proof-of-concept analysis; however, this has not been tested in real world application. Models deployed at point of care could also be used to receive feedback from the nurses and clinicians to serve as refined input to retrain the model.

## Conclusions

This proof-of-concept analysis demonstrated the potential of the creation and deployment of a real-time predictive model based on patient and dialysis treatment data. The mechanics for triggering a model endpoint based on real-time message capture and to produce real-time reporting that includes treatment metrics coupled with model inferences were successfully implemented. The challenge will be to scale for large amounts of data and to design appropriate interventions.

## Data Availability

The datasets and coding utilized for this study are not publicly available. The datasets were obtained from the Fresenius Medical Care North America Knowledge Center Data Warehouse, which is restricted to use by only authorized employees and is not publicly available.

## Abbreviations

OSD: Optical Sensing Device
ML: Machine Learning
AUROC: Area Under the Receiver Operating Curve
HD: Hemodialysis
IDH: Intradialytic Hypotension
AWS: Amazon Web Services
RBV: Relative Blood Volume
IoT: Internet of Things
HIPAA: Health Insurance Portability and Accountability Act
RTD: Remaining Time on Dialysis
MLE: Machine Learning Engine
